# A Miniaturized Platform for Multiplexed Drug Response Imaging in Live Tumors

**DOI:** 10.3390/cancers13040653

**Published:** 2021-02-06

**Authors:** Sharath Bhagavatula, Devon Thompson, Sebastian W. Ahn, Kunj Upadhyaya, Alex Lammers, Kyle Deans, Christine Dominas, Benjamin Ferland, Veronica Valvo, Guigen Liu, Oliver Jonas

**Affiliations:** 1 Department of Radiology, Brigham and Women’s Hospital, Harvard Medical School, 75 Francis Street, Boston, MA 02115, USA; devon.thompson96@gmail.com (D.T.); wahn1@bwh.harvard.edu (S.W.A.); kunj.upadhyaya@gmail.com (K.U.); lammers@bu.edu (A.L.); kyle_deans@dfci.harvard.edu (K.D.); cdominas@bwh.harvard.edu (C.D.); vvalvo@bwh.harvard.edu (V.V.); gliu19@bwh.harvard.edu (G.L.); 2 Department of Pathology, Brigham and Women’s Hospital, Harvard Medical School, 75 Francis Street, Boston, MA 02115, USA; bferland@bwh.harvard.edu

**Keywords:** personalized oncology, miniaturized optical imaging, drug screening

## Abstract

**Simple Summary:**

We have developed an implantable microdevice that is placed into a live tumor, and can directly image how effective various chemotherapy drugs are at inducing cell death, without having to remove or process the tumor tissue. Currently drug optimization is performed by assessing tumor shrinkage after treating a patient with systemic doses of a chemotherapy agent; this only evaluates a single treatment at a time and typically takes weeks-months before an optimal treatment strategy is found (if found at all) for a specific patient. In contrast, using the technology presented here, a personalized cancer treatment strategy can potentially be optimized and tailored to a specific patient’s tumor characteristics within several hours, without requiring surgical tissue removal or prolonged trials of potentially ineffective chemotherapies.

**Abstract:**

By observing the activity of anti-cancer agents directly in tumors, there is potential to greatly expand our understanding of drug response and develop more personalized cancer treatments. Implantable microdevices (IMD) have been recently developed to deliver microdoses of chemotherapeutic agents locally into confined regions of live tumors; the tissue can be subsequently removed and analyzed to evaluate drug response. This method has the potential to rapidly screen multiple drugs, but requires surgical tissue removal and only evaluates drug response at a single timepoint when the tissue is excised. Here, we describe a “lab-in-a-tumor” implantable microdevice (LIT-IMD) platform to image cell-death drug response within a live tumor, without requiring surgical resection or tissue processing. The LIT-IMD is inserted into a live tumor and delivers multiple drug microdoses into spatially discrete locations. In parallel, it locally delivers microdose levels of a fluorescent cell-death assay, which diffuses into drug-exposed tissues and accumulates at sites of cell death. An integrated miniaturized fluorescence imaging probe images each region to evaluate drug-induced cell death. We demonstrate ability to evaluate multi-drug response over 8 h using murine tumor models and show correlation with gold-standard conventional fluorescence microscopy and histopathology. This is the first demonstration of a fully integrated platform for evaluating multiple chemotherapy responses in situ. This approach could enable a more complete understanding of drug activity in live tumors, and could expand the utility of drug-response measurements to a wide range of settings where surgery is not feasible.

## 1. Introduction

Understanding the effect of anti-cancer drugs on the tumor and its microenvironment is central to the design of effective single agent and combination regimens. Currently available measures of drug response, such as tumor shrinkage, often take weeks to months to manifest and are not always representative of a tumor’s dynamic sensitivities [1,2]. An implantable microdevice (IMD) has been developed which could enable a clinician to screen the efficacy of multiple drugs or drug combinations for a specific tumor in a specific patient [3]. The IMD is implanted into a live tumor and delivers microdoses of drugs into tiny spatially discrete tissue volumes. After allowing the drug to interact with the tumor in its native microenvironment, the IMD and surrounding tissue are surgically removed, processed (e.g., formalin-fixed, paraffin embedded, sectioned, stained), and imaged using benchtop microscopy to evaluate cell death response. This method has been shown to predict and optimize cancer treatments in numerous preclinical models [3,4,5] and is currently undergoing clinical investigation in several first-in-human studies [6,7]. One limitation of this approach is that it requires surgical removal of tissue to allow quantification of cell death caused by each drug. This can be difficult or impractical for many patients unless they are already planned for cytoreductive surgery. In addition, this analysis only evaluates tissue response at a single timepoint; delayed or serial assessment is not feasible once the tissue is surgically removed.

An alternative approach is to image drug response directly in live tumors (in situ) without requiring surgical removal or tissue processing. Recent advances in fluorescence microscopy tools have enabled imaging of biological processes via miniaturized imaging probes placed directly into native tissues [8,9,10]. In addition, fluorescent assays are capable of detecting cell death and other physiologic processes within viable cell samples without the need for extensive tissue processing [11,12,13,14]. For example, propidium iodide (PI) is a fluorescent marker that is a well-established and accepted method to evaluate cell death [15,16]. PI binds double stranded DNA, but can only enter cells that have disrupted cell membranes; therefore, it accumulates in late apoptotic and necrotic cells that have lost membrane integrity. There is potential for implantable microdevices, miniaturized imaging probes, and live cell assay technologies to be combined to create ‘lab-in-a-tumor’ systems capable of in situ drug response assessment. Such systems can be used in pre-clinical settings to monitor dynamic responses to novel treatment candidates in native microenvironments, or potentially translated to a clinical setting to identify the most effective drug treatments for a specific patient’s tumor.

**Figure 1:**
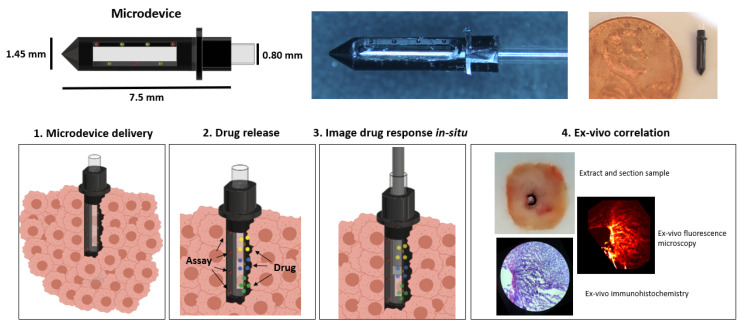
Schematic (top left) and actual image (top center and top right) of the lab-in-tumor implantable microdevice (LIT-IMD) containing multiple reservoirs for drug and assay release and a hollow inner lumen with optical window face. Bottom row demonstrates overall functionality: 1—LIT-IMD is implanted into a live tumor; 2—LIT-IMD releases multiple drugs into discrete confined tumoral tissue and in parallel, releases fluorescent cell death assay into drug-exposed tissue; 3—fluorescence imaging probe is passed coaxially into the LIT-IMD for real-time evaluation of drug diffusion and cell death response; 4—correlation was performed to validate this method by removing and sectioning tissue at each drug-exposed site and comparing in situ fluorescence imaging signal with conventional benchtop fluorescence microscopy and immunohistochemistry of processed tissue sections.

Here, we describe the first implementation of such a system. Our “lab-in-a-tumor” implantable microdevice (LIT-IMD) is placed into a tumor and locally delivers microdoses of multiple drugs, along with a local microdose of PI (Figure 1). The PI preferentially accumulates in non-viable tissues, resulting in strong fluorescent signal at the sites where drugs have effectively killed tumor tissue. A miniaturized fluorescence imaging probe is passed coaxially into the LIT-IMD, and determines the fluorescent signal from each drug exposed tissue region, resulting in a visual ‘readout’ of relative drug efficacy. We demonstrate proof-of-concept of this method to image the response to three commonly used United States Food and Drug Administration (FDA)-approved anti-cancer drugs in live murine tumors. This approach could enable serial assessment of drug diffusion and cell death response over time, without requiring tissue removal and processing. It also serves as a proof-of-concept of in situ fluorescence microscopy with local assay delivery, with potential for broad pre-clinical and clinical application.

## 2. Results

### 2.1. Design and Development of the LIT-IMD Probe

The LIT-IMD prototype is illustrated in Figure 1. It is a 7.5 mm long, 1.45 mm diameter cylinder with a 0.8 mm hollow inner lumen and a tapered conical distal end. A 5 mm long, 600 μm wide clear rectangular slot is incorporated into one side of the microdevice body, and serves as an optical window through which images of adjacent tissue can be acquired.

In total, three 200 μm diameter, 200 μm deep drug release reservoirs, or ‘micro-holes’, are machined into one wall of the LIT-IMD immediately adjacent to the optical window. These drug reservoirs are spaced 1.1 mm apart along the length of the LIT-IMD. Drug formulations are loaded into these reservoirs in solid powder form as described in Methods and are passively released into spatially distinct microscopic regions of tissue after tumor implantation. The reservoir dimensions and spacing and are optimized based on prior studies evaluating drug diffusion [3,4,17], with sufficient separation to ensure that there is no spatial overlap between drug release sites.

Additionally, four additional reservoirs are placed in parallel on the other side of the optical window, 1.1 mm apart and with 550 μm longitudinal offset from the reservoirs on the drug release side. These four reservoirs are for propidium iodide (PI) assay loading and release, with spacing optimized to allow equal assay diffusion into each drug release site. Both the drug and assay release reservoirs are angled inward 45 degrees such that they diffuse into tissue directly in front of the optical window. Therefore, a coaxially placed side-viewing imaging probe imaging in a radially outward direction can evaluate fluorescence signal within drug-exposed tissue.

### 2.2. Miniaturized 2-Color Fluorescence Microscopy (M-2CFM) System Development

#### 2.2.1. Customization and Set-up

**Figure 2:**
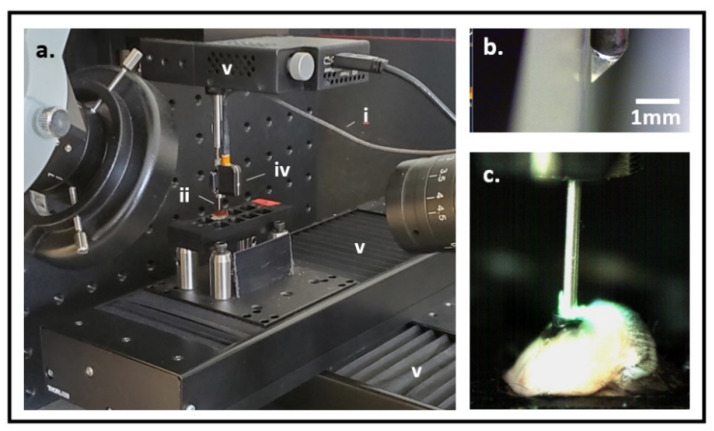
(**a**) Experimental set-up for live tissue fluorescence imaging. An optical fiber (i) delivers light excitation through a gradient index (GRIN) lens imaging probe (ii) into a tissue sample (not shown). The resultant fluorescent signal is captured by an external charge-coupled device (CCD) optical detector (iv) for 2-color fluorescence imaging. 4-axis (x, y, z linear and rotational) stage system (v) allows precise positioning of the imaging probe within the tissue sample. (**b**) GRIN lens imaging probe tip with triangular prism for side-viewing. (**c**) Imaging probe is placed through the microdevice into live murine tumoral tissue to image fluorescent drug and assay signal.

A miniaturized 2-color fluorescence microscopy (M-2CFM) system (Doric Lenses Inc., Quebec, CA, Canada) was customized to interface with the microdevice to image live tumoral tissue (Figure 2). The system detects fluorescent signals in the red (630 nm wavelength) and green (530 nm wavelength) channels. Fluorescence imaging is performed via an optical fiber coupled to a custom miniaturized 700-μm diameter side-viewing imaging probe, which can be passed coaxially within the LIT-IMD to image tissue next to the microdevice. Further details for customization of this imaging system can be found in the Methods section.

#### 2.2.2. Imaging System Characterization

**Figure 3:**
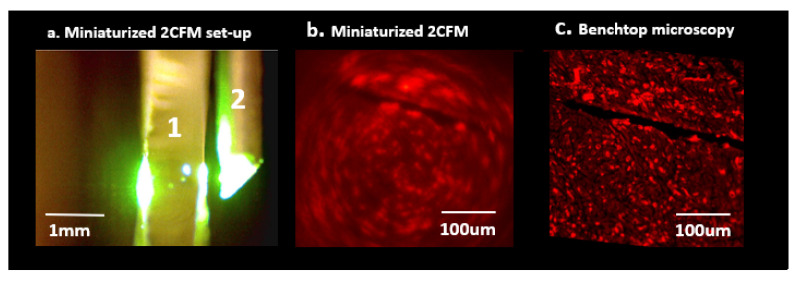
(**a**) Miniaturized 2-color fluorescence microscopy (M-2CFM) of stained tissue sections placed on a slide (1) using a side-imaging GRIN lens probe (2). (**b**) M-2CFM of red-fluorescent labeled macrophages. (**c**) Corresponding benchtop fluorescence image of the same slide used to quantify M-2CFM imaging capabilities. In-plane resolution was sufficient to view individual 10 μm diameter cells, and overall field of view was ~400 × 400 μm.

Performance of the customized M-2CFM imaging system was evaluated using a 10-μm thick tissue section containing fluorescently stained macrophage cells (Figure 3). The optimal working distance of the M-2CFM system was 200 μm and the field of view was ~400 × 400 μm. The in-focus resolution was sufficient to detect individual cells as small as 10 um in diameter, based on correlation with a benchtop fluorescence microscope system (Figure 3b,c). Fluorescent signal remained visible at up to 300 μm distance from the probe. Therefore, in bulk tissues, the received images represent an accumulated effect of fluorescent signal excited within a depth of ~300 μm of the probe.

**Figure 4:**
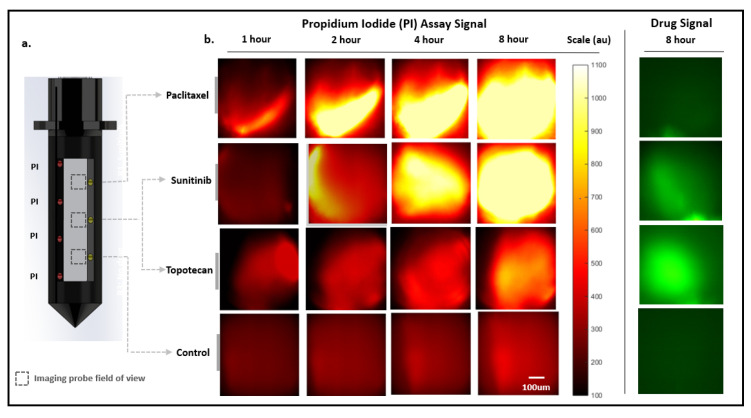
Miniaturized 2-color microscopy (M-2CFM) over 8 h in a live murine MC38 tumor. (**a**). Microdevice drug and assay loading diagram. The three distinct drug release sites spaced 1.1 mm apart contain Paclitaxel (*n* = 5), Sunitinib (*n* = 3) or Topotecan (*n* = 2), and Control (inert polyethylene glycol, PEG) (*n* = 5). Propidium iodide (PI) assay is loaded and released from four reservoirs located on the opposite side of the microdevice optical window. PI diffuses equally into all drug-exposed tissue regions (dotted boxes), which can be individually imaged. (**b**). M-2CFM fluorescent imaging of each drug-exposed tissue region in red (assay) and green (drug) channels over 8 h.

Although the imaging system had high in-plane resolution capable of detecting individual cells on a microscope slide, high resolution distinction of individual cells or tissue structure in bulk tumor tissue was not possible, likely due to the lack of ‘optical sectioning’ (limiting the optical input to a single plane at the optimal focal distance). Therefore, in situ bulk tumor tissue images were composites of overlapping fluorescent signal from multiple tissue planes, resulting in ‘blurred’ images representing total fluorescence within a 3-dimensional ~400 × 400 × 300 μm drug-exposed volume of tissue to the side of the imaging probe (Figure 4).

### 2.3. Drug Response Assessment Proof-of-Concept in Murine Tumor Model

#### 2.3.1. Serial Miniaturized 2-Color Fluorescence Microscopy (M-2CFM) of Drug Diffusion and Response

In total, five LIT-IMDs, each preloaded with three drugs and PI assay as shown in Figure 4a, were placed into five separate murine MC38 subcutaneous tumors. The drugs were reconstituted with polyethylene glycol (PEG) and loaded in a solid-powder form. The drug formulation was selected in a manner that has been previously shown to contain local drug diffusion to within 100–400 μm radially of the drug release sites, and has been shown to approximate systemic dosing at these depths (see Methods) [3,4,5,17]. Specific drug responses to three chemotherapy agents could be tested in each tumor, since each device had three unique drug release sites. A total of five tumors containing fifteen unique drug release sites were evaluated in total. In three of the tumors, we evaluated response to Paclitaxel, Sunitinib, and Control (inert PEG) agents. In two of the tumors, we evaluated response to Paclitaxel, Topotecan, and Control.

Since our method did not require tissue sectioning or processing, we were able to assess drug diffusion and response serially over time. We monitored drug diffusion in the green channel for Sunitinib and Topotecan, which are intrinsically fluorescent. As expected, both drugs remained spatially confined to less than 500 μm of the drug release sites at 8 h in all tumors. Importantly, there was no spatial overlap of drugs into adjacent drug release sites.

We serially imaged PI accumulation in the red channel to compare cell death drug response over time at each of the drug-exposed tissue sites (Figure 4). As described above, the measured signal represented overall PI accumulation within an ~400 × 400 × 300 μm volume of drug exposed tissue at each drug release site. Although evaluation of PI uptake within individual cells was not possible with this imaging system, the cumulative fluorescent signal at each site provided an overall assessment of cell death within each volume of drug-exposed tissue.

**Figure 5:**
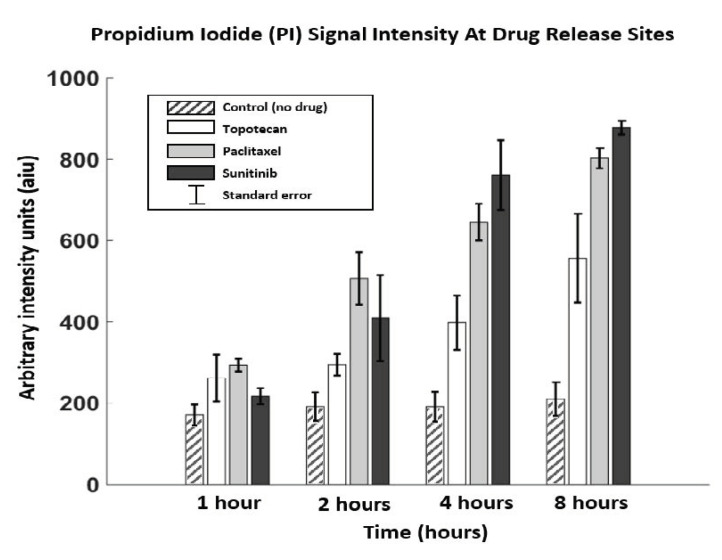
Mean and standard error (SEM) signal intensities obtained at each time point for each drug and control. All three drug sites had significantly higher PI signal compared to the Control site, indicating drug response (*p* < 0.001). Both Sunitinib and Paclitaxel had higher signal at 8 h compared to Topotecan [p(sun vs. top) = 0.003; p(pac vs. top) = 0.01], which suggests higher therapeutic response at the delivered drug concentrations.

Summary data from serial assessment in 5 replicate tumors and 15 drug sites is presented in Figure 5 and Table 1. There was significant PI signal increase at Paclitaxel and Sunitinib drug sites over time (ANOVA F(3, 10) = 34.0, *p* < 0.001 for Paclitaxel and F(3, 5) = 27.3, *p* = 0.002 for Sunitinib), with PI signal saturation at 8 h indicating very high assay accumulation and cell death at both drug sites. PI signal at Topotecan and Control sites increased over time but did not reach significance (F(3, 4) = 3.44, *p* = 0.13 for Topotecan; F(3, 10) = 0.23, *p* = 0.87 for Control). None of the drug sites were significantly higher than Control at 1 h, but all were higher than control by 8 h (see Table 1 for mean intensity and *p*-values values). There were no significant differences in signal intensity in comparing Paclitaxel vs. Sunitinib (*p* = 0.53 at 8 h); however, both Paclitaxel and Sunitinib had higher signal intensity compared to Topotecan (p(Paclitaxel vs. Topotecan) = 0.01; p(Sunitinib vs. Topotecan) = 0.003).

These findings overall indicate a higher efficacy to induce tumor cell death of Sunitinib and Paclitaxel in the tested MC38 tumors compared to Topotecan, and as expected, higher tumor cell death for all tested chemotherapeutic agents compared to Control. The baseline PI signal at the Control site is likely from passive PI diffusion and physiologic tumoral cell death; the additional signal at the drug sites compared to control likely indicate PI accumulation from drug-induced cell death. These results were confirmed by correlating with gold standard ex vivo analysis as described below.

#### 2.3.2. Correlation with Gold-Standard Cell Death Assessment Methods

**Figure 6:**
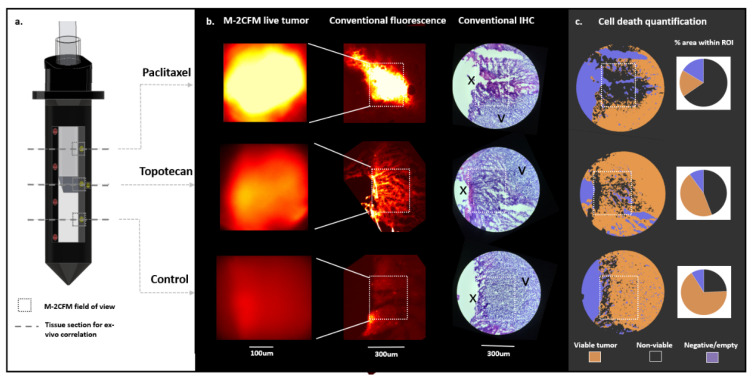
Methodology to correlate live tumor imaging with gold standard ex vivo fluorescence imaging and immunohistochemistry, demonstrated in a representative tumor sample. (**a**) After completion of live tumor imaging for 8 h, cross sections of tumor tissue (dashed lines) were obtained at each drug delivery site. (**b**) fluorescent signal from M-2CFM imaging in live tumor tissue (first column) was compared with corresponding conventional benchtop fluorescence microscopy (second column) and immunohistochemistry (IHC) (third column), imaged after sectioning and processing the tissue. On the benchtop fluorescence and IHC images, the boxes indicate the ~400 × 300 μm region of interest (ROI) corresponding to the region of tissue imaged by the M-2CFM probe in the live tumor. On the IHC image, the ‘X’ denotes site of drug release; the ‘V’ indicates viable tumor tissue. (**c**) A trained image classifier enables quantitative determination of a non-viability index, NVI (non-viable divided by viable tumor) from the stained IHC images, which is then correlated with the experimental fluorescence images.

**Figure 7:**
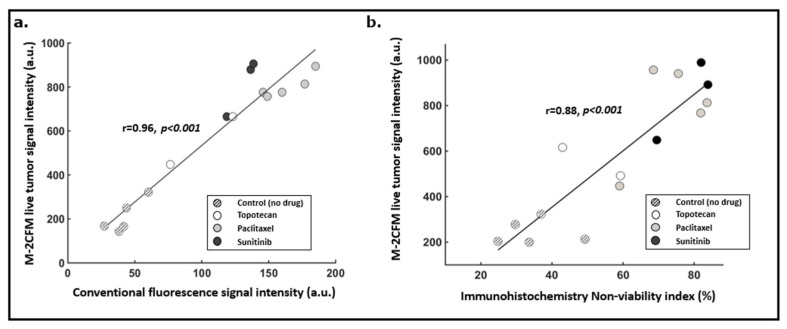
(**a**) Correlation between the M-2CFM live tumor fluorescence imaging and conventional benchtop fluorescence imaging of corresponding tumor sections (r = 0.96, *p* < 0.001). (**b**) Correlation between the M-2CFM live tumor fluorescence imaging signal intensity and a non-viability index calculated from gold standard immunohistochemistry hematoxylin and eosin (H&E) stained sections (r = 0.88, *p* < 0.001). *n* = 15 replicate drug sites from five tumors were used for this correlation.

Current gold-standard methods to evaluate cell death include conventional benchtop fluorescence imaging of propidium iodide and immunohistochemistry (IHC) analysis. IMD-based drug screening using IHC cell death quantification of resected drug-exposed tumor samples has been previously shown to be predictive of systemic response [3]. Therefore, if our live tumor imaging approach correlates strongly with IHC-based assessment of cell death, this would indicate that it can be used for IMD-based drug screening in lieu of IHC. Correlation with benchtop fluorescence imaging and IHC was performed in 15 distinct replicate drug-exposed tissue regions (methodology illustrated in Figure 6 and further described in Methods). We observed a strong correlation with both benchtop fluorescence microscopy (Pearson r = 0.96, *p* < 0.001) and immunohistochemistry (Pearson r = 0.88, *p* < 0.001) (Figure 7). The in situ fluorescence signal measured within each drug-exposed tissue volume increased linearly with the overall percent of non-viable tissue within that same region as assessed by IHC (Figure 7).

These findings indicate that in situ measurement of PI signal intensity using the LIT-IMD is representative of overall cell death and nonviability within a drug exposed tissue volume, and therefore could obviate the need for ex vivo tissue processing and IHC analysis for IMD-based drug screening.

## 3. Materials and Methods

### 3.1. Design and Development of the LIT-IMD Probe

The body of the LIT-IMD is made from Delrin acetyl-resin (McMaster–Carr). Solidworks (Dassault Systems, Solidworks 2017) and Mastercam (CNC Software, Inc., Tolland, CT, USA) was used to develop CAD and CAM designs of the device body and a CNC milling machine (MDA Precision, TN5-V8-TC8) was used to fabricate the part from the Delrin stock material. To protect the optical probe from contamination when placed into the LIT-IMD, a silica capillary tube with an outer diameter of 0.8 mm (Charles Supper Company, Natick, MA, USA) is epoxied to the inner surface of the microdevice body to form a water-tight inner lumen for coaxial passage of the miniaturized fluorescence imaging probe.

### 3.2. Miniaturized 2-Color Fluorescence Microscopy (M-2CFM) System Development

#### 3.2.1. System Customization and Set-up

The M-2CFM system (Doric Lenses Inc., Quebec, CA, Canada) consists of a broad band Ce:YAG light source and a blue LED source centered at 465 nm. The spectrum of the Ce:YAG source is filtered by a narrow bandpass optical filter centered at 561 nm. The two wavelengths or colors thus function as distinct excitation channels which can be controlled separately. The two colors are combined and coupled into a 200 μm core diameter multimode optical fiber. Light excitation for imaging is transmitted via an optical fiber coupled to a customized thin 1 cm long, 500 μm diameter cylindrical gradient index (GRIN) lens imaging probe, which fits coaxially into the LIT-IMD inner lumen. A metal sheath with 700 μm outer diameter and 100 μm wall thickness mechanically enhances and protects the probe. To enable side-viewing necessary for our application, the GRIN lens is coupled to a triangular prism at its distal end which redirects light 90 degrees (Figure 2b).

The probe both delivers light excitation and also collects the resultant fluorescent signal. The system operates in an epi-fluorescence manner, and the collected fluorescence images are received by two separate CCD cameras for both channels, one centered at 630 nm (red channel) and the other at 530 nm (green channel). The miniaturized optical imaging probe is mounted on a 4-axis stage system consisting of three linear stages (Thorlabs) for precise X-Y-Z movement and a fourth rotational stage (Figure 2a). The stages are controlled electronically using commercially available software (Thorlabs). Control of the M-2CFM system and subsequent imaging display from the CCD camera are also performed in real-time using commercially available software (Doric). There were two microscope cameras (Motic Instruments, Schertz, TX, USA) used to confirm satisfactory positioning and alignment of the imaging probe, and to ensure that the microdevice does not migrate within the live tumoral tissue during imaging.

#### 3.2.2. Imaging System Characterization

A 10-μm thick section containing fluorescently stained macrophage cells was placed on a slide. The slide was moved in 25 μm increments away from the imaging probe using a linear stage (Thorlabs) until optimal focus and minimal blurring was qualitatively achieved; this distance was determined to be the optimal ‘working distance’ for the system. Images obtained using the M-2CFM system were compared to gold standard benchtop fluorescence imaging (Echo Revolve, San Diego, CA, USA) to determine the overall field of view and resolution of the M-2CFM system with the custom GRIN lens probe.

### 3.3. Drug Response Assessment Proof-of-Concept in Murine Tumor Model

#### 3.3.1. Animal Model

Institutional animal care and use committee (IACUC) approval was obtained. Murine subcutaneous MC38 (colon adenocarcinoma) tumors were used for this study. Approximately 200 μL cell suspension (10 × 106 cells/mL) was injected into the subcutaneous murine flank region under 1–3% isoflurane anesthesia, and tumors grew to 1.5 cm maximal diameter prior to microdevice insertion. In total, five replicate murine tumors each containing one LIT-IMD device were used, for a total of 15 unique drug-exposed tissue regions. Microdevices preloaded with drug and assay were directly inserted into live tumoral tissue. After IMD implantation, the preloaded microdoses of drugs passively diffused into spatially discrete tissue regions. The PI assay microdoses also passively diffused into these drug-exposed tissue regions.

#### 3.3.2. Drug and Assay Formulation for Localized Delivery

Chemotherapeutic agents Paclitaxel, Sunitinib, and Topotecan were used for our live tumor experiments. All drugs were purchased in solid powder form (Selleck Chem, Houston, TX, USA) and reconstituted with 1450 g/mole molecular weight polyethylene glycol (PEG) (Alfa Aesar, Haverhill, MA, USA) into a 50% *w/w* drug-PEG-1450 composite powder.

Propidium iodide (PI) was also purchased in powder form (Sigma–Aldrich, St. Louis, MO, USA) and reconstituted with PEG-1000 into a 75% *w/w* assay-PEG composite powder. A lower concentration and lower molecular weight PEG was used for the PI formulation compared to the drugs, which allowed greater spatial diffusion of assay material into adjacent drug-exposed tissue sites.

The chemotherapy agents were loaded into the microdevice in consecutive reservoirs as shown in Figure 4a. The PI assay formulation was loaded on the opposite wall of the microdevice into four contiguous reservoirs (Figure 4a). This formulation and loading scheme optimized equal diffusion of PI into each drug-exposed tissue region, upon delivery of the LIT-IMD into live tumor tissue.

M-2CFM imaging at each drug release site within the tumor was performed with the imaging probe left within a tumor for 8 h after microdevice implantation. Images at each drug delivery site were obtained at 1, 2, 4, and 8 h timepoints (Figure 4b). Fluorescence images in the green (drug) and red (assay) channels were obtained using identical imaging parameters at each drug release site: exposure time 100 ms; LED 465 nm excitation and Ce:YAG laser 561 nm excitation. For each image, the average signal intensity within the field of view was measured as quantitative indicator of overall drug diffusion (green channel) and cell death (red channel), respectively.

Red (assay) channel intensity was presumed to represent cell death from PI binding. We performed statistical analysis comparing cell death at each drug site in the tumors at each time point over 8 h. This assessment was done for three drugs and one control site in 5 tumors (*n* = 5). We present mean and standard error of mean values for each site at each time point (Table 1 and Figure 5). A one-way analysis of variance (ANOVA) test was used to compare signal means. The *p*-values listed in the table are pairwise comparisons of drug exposure sites relative to the control site and indicate the degree of certainty that a significant difference in cell death is observed relative to control.

#### 3.3.3. Tissue Processing for ex vivo Correlation

At 8-h after LIT-IMD implantation, the tumors were immediately frozen at −80 degrees with the microdevices in place. After embedding into optimum cutting temperature (OCT) media, 10-μm sections were obtained at each drug site perpendicular to the long axis of the LIT-IMD using a cryostat system (Leica, Buffalo Grove, IL, USA) (Figure 6a). Hematoxylin and eosin (H&E) staining was performed of the same or immediately adjacent sections for immunohistochemistry (IHC) analysis.

#### 3.3.4. Conventional Imaging and Image Analysis

Conventional fluorescence imaging of the processed tissue sections was performed as a gold standard measurement in the red and green channels using a commercial benchtop fluorescence microscope system (Echo Revolve, San Diego, CA, USA). Images for analysis were obtained at 4X magnification with 300 ms exposure time in red (586 nm wavelength excitation, 603 nm emission) and green (495 nm excitation, 519 nm emission) channels. A 400 × 300 μm rectangular region of interest (ROI) was selected at each drug site, corresponding to a representative area evaluated by M-2CFM system in the live tumor (dotted white box in Figure 6b). The mean PI (red channel) signal intensity within the ROI was calculated using Matlab image processing software (Mathworks), as a quantitative measure of cell death.

Hematoxylin and eosin (H&E) stained immunohistochemistry (IHC) slides were imaged at 10X magnification. A 400 × 300 μm ROI at each drug release site was selected in a similar manner as described above. Using QuPath software (QuPath) [18], an image classifier was trained and validated, as described in Section 3.3.5, and used to segment regions of viable and non-viable tumor. A non-viability index (NVI) was calculated at each site as a quantitative measure of cell death: NVI = (non-viable tumor area)/(total viable and non-viable tumor area) × 100. The NVI was correlated with the corresponding mean M-2CFM signal intensity.

Correlation between M-2CFM vs. benchtop fluorescence ROI signal intensities and M-2CFM vs. IHC-based NVI were calculated using Matlab. For this analysis, we quantified cell death drug response with each method at 15 distinct tumor sites (*n* = 15) in five different tumors. Pearson correlation tests were performed, and correlation coefficients (r) and significance values (*p*) are reported (Figure 7).

#### 3.3.5. Automated Image Classification

Hematoxylin and eosin (H&E)-stained slides from separate MC38 murine tumors were used for training, validation, and final analysis of the IHC images. The training set consisted of 64 spatial regions of interest (ROI) in 10 separate tumors, with the total training set consisting of 770,304 pixels and 1472 cells. The ROIs were pre-annotated as viable, negative or empty space, or non-viable (based on standard pathological measures of necrosis and late apoptosis, including cell membrane disruption, loss of cellular architecture, cell shrinkage, and nuclear condensation and fragmentation, as reviewed with a clinical pathologist). QuPath [18], a publicly available software platform for immunohistochemistry analysis, was used to train an image classifier based on the annotated training set. An artificial neural network model was used for this classifier.

The model was validated on a separate MC38 tumor validation set consisting of 55 ROIs containing 665,500 pixels and 1265 cells. Each ROI was pre-defined as viable, negative, or non-viable based on gold-standard pathology evaluation and compared with the classifier prediction (See Supplementary Figure S1). For non-viable, negative, and viable classes, respectively, the per-class precision was 0.92, 1.00, and 0.94; recall 0.96, 1.00, 0.88; and F1-score 0.94, 1.00, 0.91. The macro-averaged precision, recall, and F1-score was 0.95, 0.94, and 0.95, respectively. This classifier was applied to experimental data images, which were obtained using the same staining protocol and imaging parameters as the training and validation sets (Figure 6 and Supplementary Figure S2).

## 4. Discussion

This study demonstrates that localized drug-induced cell death can be assessed directly in a live tumor, and represents a unique approach compared to current histopathology methods that require tissue removal and processing. This could enable IMD-based drug screening to be performed more rapidly and efficiently, as there is no delay associated with tissue processing, staining, and ex vivo analysis required for current IHC methods. Since imaging is performed directly within the tumor at the precise site of drug release, drug response assessment can be performed in tumors of any size, as long as the tumors are large enough for the microdevices to be placed inside of them. In our study, we used tumors of 1–1.5 cm maximal diameter, but our method would be applicable even in much larger tumors. In addition, since tissue can be analyzed in place without need for surgical removal, this approach may enable IMD-based drug screening and other similar applications to be more safely implemented in a greater number of pre-clinical and clinical settings.

The current system was optimized for use in superficial murine tumors, and can be used in its current design form for similarly superficial skin or subcutaneous tumors. The small size and cylindrical shape of the microdevice also makes it potentially suitable for minimally invasive implantation in deeper tissues, using a procedure similar to percutaneous fiducial marker delivery [19]. A potential design strategy for imaging deeper tumors would be to place the LIT-IMD at the end of a longer imaging catheter, similar to currently existing endoluminal and endovascular catheters [20,21]. This could further increase the number of pre-clinical and clinical settings for which IMD-based drug screening is safe and feasible.

The M-2CFM system imaged drug-induced cell death with strong differences in signal intensity at sites of cell death compared to sites of viable tumor. However, overlap of fluorescence signal detection from in-focus and out-of-focus tissue planes limited overall resolution. Miniaturized confocal and multiphoton microscopy systems are available that could allow higher resolution and optical sectioning, wherein a specific tissue plane at a focal distance from the imaging probe is imaged without signal overlap from out-of-focus planes [9,22,23]. However, these are currently limited by low penetration depth and, therefore, less suitable for our application, which benefits from volumetric assessment of drug exposed tissue up to 300 μm depths from the imaging probe. Further development of miniaturized confocal or multiphoton fluorescence imaging with larger penetration depth could enable higher resolution and optical sectioning in deeper tissues in the future. Label-free microscopy systems such as optical coherence tomography and Raman spectroscopy also could have the potential to quantify cell death in situ [24,25,26], but have not been extensively studied or validated.

Local delivery of PI assay microdoses enabled detection and comparison of cell death response among multiple drugs. With this approach, both drug and assay are confined to tiny tumoral tissue volumes, obviating potential off-target toxicity from systemic (e.g., intravenous or oral) administration. Other existing targeted fluorescent imaging probes can be used in a similar manner to more fully evaluate biological mechanisms of disease and treatment response. Existing fluorescent probes can detect numerous biological processes including apoptosis, immune response, and genetic expression in live tissues [12,27,28]. However, other than a few non-targeted agents such as indocyanine green and fluorescein [20,29,30,31], the vast majority of fluorescent imaging probes are not FDA-approved for clinical use and have not been used in patients due to concerns for off-target toxicity with systemic delivery. Restricting the delivery of these agents to microdoses released locally adjacent to the LIT-IMD, as described here for PI, eliminates systemic toxicity risk. This approach could therefore enable more widespread use of labeled in vivo fluorescence microscopy in preclinical and clinical settings. Although we have demonstrated utility specifically for cell death drug response, there is potential for this platform to be broadly applied to monitor a diverse range of biological processes and individual resistance mechanisms in native tumoral microenvironments.

This is the first study to our knowledge in which the three tested drugs (Paclitaxel, Sunitinib, Topotecan) were directly compared in a syngeneic live tumor mouse model. The relative effectiveness measured in our microdose study in MC38 tumors are congruent with prior in vivo and in vitro published results. Previous systemic in vivo studies have shown MC38 murine models to exhibit significantly greater sensitivity to Paclitaxel than other commonly used tumor models [32]. Paclitaxel has been shown to be more effective than Topotecan in MC38 organoid and slice culture models [33]. Though Sunitinib has not been tested in MC38 previously, highly similar drugs that also target vascular endothelial growth factor (VEGF) and platelet-derived growth factor recepter (PDGFR) such as Vandetanib have shown to be highly potent in MC38 tumors [34]. High-throughput in vitro sensitivity results that are part of the Sanger and Cell Line Encyclopedia in MC38 and other colorectal cell lines with similar mutational profiles (e.g., COLO201, COLO205) also indicate that VEGF and PDGFR inhibitors such as Sunitinib are the most potent (z-score −0.84), closely followed by Paclitaxel (z-score −0.79), with Topotecan being significantly less potent (z-score +0.77) [35].

Intratumoral heterogeneity remains a significant challenge in developing personalized cancer treatment strategies [36,37]. Diagnostic and molecular information ascertained from a small tumor sample may not be representative of the entire tumor, leading to suboptimal treatments. Our platform could simultaneously evaluate drug response at multiple spatially discrete regions of a tumor, either by redundant release of the same drug into multiple sites from a single microdevice or by placing multiple identically loaded microdevices into different tumor regions. While tumor biopsies are often limited by the amount of tissue that can safely be removed and assessed, full in situ assessment may allow interrogation of a greater volume of tissue and more fully evaluate heterogeneous drug response. However as even this approach does not evaluate the entire tumor, it may remain susceptible to the limitations of intratumoral heterogeneity. Therefore, additional studies are needed to evaluate the ability to predict systemic treatment response in the setting of tumor heterogeneity.

## 5. Conclusions

Our platform enables multiplexed drug response assessment directly in live tumors without requiring surgical tissue excision, which could lead to development of more optimized treatment strategies in pre-clinical and clinical settings. The combination of in situ fluorescence imaging and local fluorescent assay delivery is a powerful approach that could greatly expand ability to image biological processes in real time.

## Figures and Tables

**Table 1:** Miniature 2-color microscopy (M-2CFM) of propidium iodide (PI) at drug and control sites. Mean and standard error of the mean (SEM) are reported in arbitrary signal intensity units (aiu) obtained at each time point for each drug/control site. *p*-values indicate comparison between signal intensities at each drug site compared to Control (*p* < 0.05 was used as a measure of significance).

	Control	Topotecan (*p*-Value)	Paclitaxel (*p*-Value)	Sunitinib (*p*-Value)
**1 h**	170.9 ± 44	261.9 ± 81.3 (0.24)	293.8 ± 27.0 (0.063)	217.3 ± 27.9 (0.70)
**2 h**	191.7 ± 61.1	294.6 ± 37.7 (0.67)	507.0 ± 111.6 (0.028)	409.35 ± 149.6 (0.16)
**4 h**	191.5 ± 63.9	398.1 ± 94.5 (0.13)	645.4 ± 78.2 (0.0026)	761.2 ± 121.8 (0.0014)
**8 h**	210.1 ± 92.3	556.6 ± 154.6 (<0.001)	802.9 ± 55.1 (<0.001)	877.9 ± 28.9 (<0.001)

## Data Availability

The data presented in this study are openly available in Harvard Dataverse repository at https://doi.org/10.7910/DVN/XRYK3Z (accessed on 2 January 2021).

## Supplementary Materials

The following are available online at https://www.mdpi.com/2072-6694/13/4/653/s1, Figure S1. (a) Hematoxylin-and-eosin (H&E) stained slide of MC38 murine tumor demonstrating viable (V), non-viable (NV) tissue, and negative/empty space (N). (b) Propidium iodide staining confirms region of non-viable tissue (red fluorescent signal). (c) Trained image classifier correctly identifies the three distinct classes, which are used to calculate the non-viability index (NVI) in our study. Three example regions of interest (ROIs, red boxes) in this tumor sample are shown. 64 such ROIs were used for training the classifier, and 55 ROIs in separate tumor samples/images were used for validation (this image is part of the validation set); Figure S2. (a) Hematoxylin-and-eosin (H&E) stained sections of drug-exposed tissue in MC38 murine tumor after local release of Paclitaxel (a), Topotecan (b), and Control (c). Magnified images from Figure 6b show greatest cell death drug response for Paclitaxel > Topotecan > Control in this tumor sample.
