# Supplementary material for: A Miniaturized Platform for Multiplexed Drug Response Imaging in Live Tumors
Source: Cancers (Basel). 2021 Feb 6;13(4):653. doi: 10.3390/cancers13040653 (PMC7915324; doi:10.3390/cancers13040653)
Supplement: Supplementary file 1 [file cancers-13-00653-s001.pdf]

cancers

MDPI

Article

A Miniaturized Platform for Multiplexed Drug Response Imaging in Live Tumors

Sharath Bhagavatula [1,*], Devon Thompson [1], Wooseok Sebastian Ahn [1], Kunj Upadhyaya [1], Alex Lammers [1], Kyle Deans [1], Christine Dominas [1], Benjamin Ferland [2], Veronica Valvo [1], Guigen Liu [1] and Oliver Jonas [1,*]

Supplementary figures:

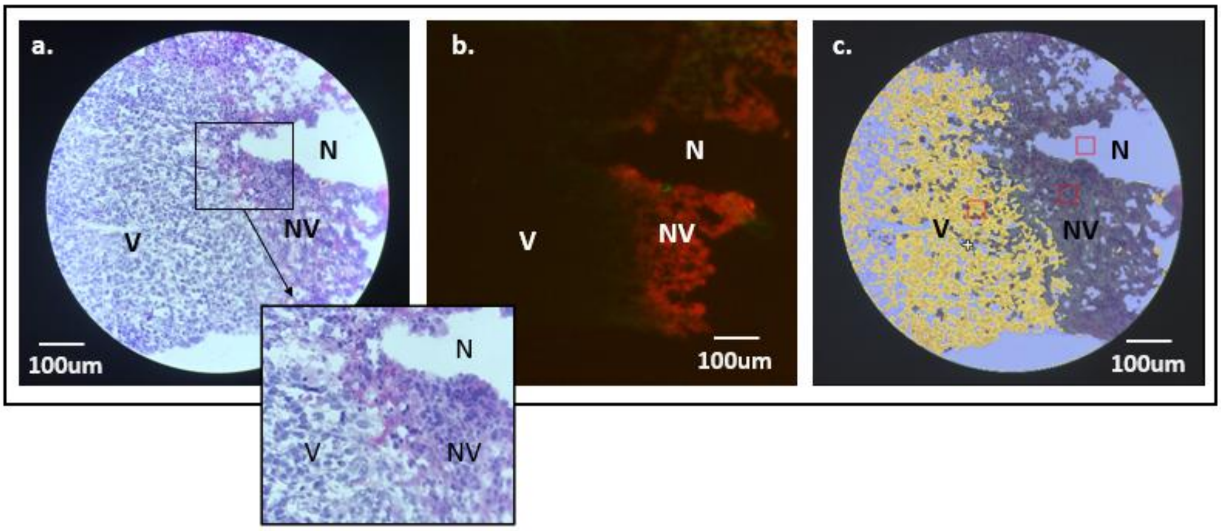

Figure S1. a) Hematoxylin-and-eosin (H&E) stained slide of MC38 murine tumor demonstrating viable (V), non-viable (NV) tissue, and negative/empty space (N). b) Propidium iodide staining confirms region of non-viable tissue (red fluorescent signal). c) Trained image classifier correctly identifies the three distinct classes, which are used to calculate the non-viability index (NVI) in our study. Three example regions of interest (ROIs, red boxes) in this tumor sample are shown. 64 such ROIs were used for training the classifier, and 55 ROIs in separate tumor samples/images were used for validation (this image is part of the validation set).

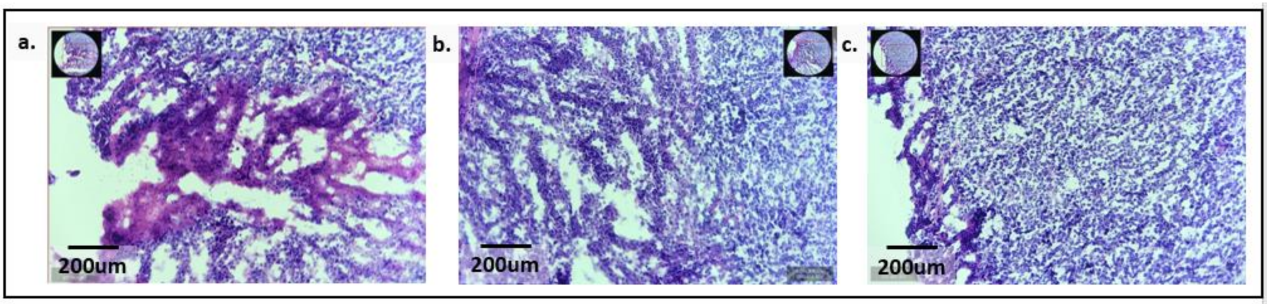

Figure S2. a) Hematoxylin-and-eosin (H&E) stained sections of drug-exposed tissue in MC38 murine tumor after local release of Paclitaxel (a), Topotecan (b), and Control (c). Magnified images from figure 6b show greatest cell death drug response for Paclitaxel > Topotecan > Control in this tumor sample.
